# Gut microbe metabolism of small molecules supports human development across the early stages of life

**DOI:** 10.3389/fmicb.2022.1006721

**Published:** 2022-09-13

**Authors:** Chiara Tarracchini, Federico Fontana, Leonardo Mancabelli, Gabriele Andrea Lugli, Giulia Alessandri, Francesca Turroni, Marco Ventura, Christian Milani

**Affiliations:** ^1^Laboratory of Probiogenomics, Department of Chemistry, Life Sciences, and Environmental Sustainability, University of Parma, Parma, Italy; ^2^GenProbio Srl, Parma, Italy; ^3^Department of Medicine and Surgery, University of Parma, Parma, Italy; ^4^Microbiome Research Hub, University of Parma, Parma, Italy

**Keywords:** metatranscriptomic, infant, gut, microbiota, development

## Abstract

From birth to adulthood, the human gut-associated microbial communities experience profound changes in their structure. However, while the taxonomical composition has been extensively explored, temporal shifts in the microbial metabolic functionalities related to the metabolism of bioactive small molecules are still largely unexplored. Here, we collected a total of 6,617 publicly available human fecal shotgun metagenomes and 42 metatranscriptomes from infants and adults to explore the dynamic changes of the microbial-derived small molecule metabolisms according to the age-related development of the human gut microbiome. Moreover, by selecting metagenomic data from 250 breastfed and 217 formula-fed infants, we also investigated how feeding types can shape the metabolic functionality of the incipient gut microbiome. From the small molecule metabolism perspective, our findings suggested that the human gut microbial communities are genetically equipped and prepared to metabolically evolve toward the adult state as early as 1 month after birth, although at the age of 4 years, it still appeared functionally underdeveloped compared to adults. Furthermore, in respect of formula-fed newborns, breastfed infants showed enrichment in microbial metabolic functions related to specific amino acids present at low concentrations in human milk, highlighting that the infant gut microbiome has specifically evolved to synthesize bioactive molecules that can complement the human breast milk composition contributing to complete nutritional supply of infant.

## Introduction

The complex microbial community associated with the human gut encompasses trillions of bacteria collectively referred to as the gut microbiota (Thursby and Juge, 2017). The process of gut microbiota establishment is reported to complete in a time window of approximately 3 years after childbirth (Palmer et al., 2007). During this period, functional capabilities of the infant gut microbiome shift from the early lactate utilization toward plant polysaccharide breakdown, vitamin biosynthesis, and xenobiotic degradation, ultimately attaining adult-like microbiome capabilities (Koenig et al., 2011; Avershina et al., 2016). However, host and environmental factors, such as dietary habits, illness, and antibiotic treatments, continue to impact and modulate the gut microbiota structure from early infancy to adulthood (Hasan and Yang, 2019).

As a large part of the microbial cells in the human gut are metabolically active, they are constantly influencing local and systemic host physiology. This ability is mediated by the production of thousands of unique bioactive small molecules, i.e., chemical compounds with a molecular weight < 3,000 Da (Pauer et al., 2021), which can accumulate in the intestine or reach organs and tissues through the blood circulatory system (Han et al., 2010; Uchimura et al., 2018; Vojinovic et al., 2019). These microbial metabolites can originate both from modifications to host-derived molecules, resulting in the production of branched- and short-chain fatty acids, secondary bile acids, and amino acids derivates such as tryptophan metabolites (Dai et al., 2011; Ridlon et al., 2014, 2016; Morrison and Preston, 2016; Liu and Dai, 2020), or from *de novo* synthesis through secondary microbial metabolism (also known as specialized metabolism), which produce a wide range of molecules such as polyketides, non-ribosomal peptides (NRPs), terpenes, NRP synthetase–independent siderophores, and saccharides (Donia and Fischbach, 2015; Postler and Ghosh, 2017). Accordingly, a large assortment of small molecules has been isolated from human gut-associated bacteria, highlighting their close involvement in host cellular functions and disease (Luber and Kostic, 2017; Bekkers et al., 2021; Tarracchini et al., 2021a). However, the small molecule repertoire from the human gut microbiota and its evolution from infancy to adulthood have been poorly explored. In this context, we exploited 6,617 publicly available shotgun metagenomic and 42 metatranscriptomic sequencing data of fecal samples from infants (aged 0–4 years) and adults to infer microbial metabolic features related to small molecules and secondary metabolites production across different stages of human life.

## Materials and methods

### Sample collection

A total of 6,617 publicly available shotgun metagenomic sequencing data of fecal samples from 4,062 infants aged 0–4 years and 2,555 adults (18–70 years) were collected from 63 different BioProjects of the Sequence Read Archive (SRA) database (Supplementary Table 1). According to the associated metadata, infants were born at term *via* uncomplicated Cesarean or natural vaginal delivery and assumed or did not breast milk (Supplementary Table 1). All subjects were overall healthy, had no history or clinical evidence of any disease, and did not take antibiotics at the time of sample collection. Similarly, 42 metatranscriptomic data from the gut microbiome of 26 infants (aged between 1 month and 1 year) and 16 adults (18–70 years) were retrieved from the public repository of NCBI (Supplementary Table 1).

### Data processing of the metagenome and metatranscriptome sequences

After being recovered from the SRA public repositories, the collected shotgun sequencing data were filtered for quality (minimum mean quality score, 20; window size, 5 bp; and minimum length, 80 bp). Subsequently, metagenomics reads aligning/mapping to the *Homo sapiens* genome sequence were identified through blastn program and removed. Taxonomic profiling of the retained sequenced reads was achieved with the METAnnotatorX2 bioinformatics platform (Milani et al., 2021), using the up-to-date RefSeq (genome) sequence database retrieved from the National Center for Biotechnology Information (NCBI).^1^ Species-level taxonomic classification of each read was achieved through Megablast (Chen et al., 2015) (with option -*e*-value 1e-5, -qcov_hsp_perc 50) using > 94% alignment identity. Reads that showed the same sequence identity against more than one bacterial species were discarded. Based on species abundance, the similarity between samples (beta-diversity) was computed using Bray-Curtis dissimilarity calculated for pairwise comparisons. Principal Coordinates Analysis (PCoA) representation of beta-diversity was performed using ORIGIN 2021.^2^

### Functional analyses based on MetaCyc database of small molecules and gene ontology enrichment

An implemented function of the software METAnnotatorX2 (Milani et al., 2021) was employed for the functional classification of the metagenome- and metatranscriptome-derived microbial reads according to the MetaCyc database of small molecules (Caspi et al., 2018). Subsequently, for each of the profiled microbial enzymes involved in small molecule metabolism, we collected the associated GO terms (all parentals) through the Bioconductor annotation data package GO.db (release 3.15).^3^

### Statistical analyses

The software SPSS version 25, and ORIGIN version 9.8.0.200^4^,^5^ were used for statistical data analyses and graphing. Principal Coordinates Analysis (PCoA) based on Bray-Curtis dissimilarity matrix was performed using the software QIIME 2 (Bolyen et al., 2019). PERMANOVA analyses were conducted using 1,000 permutations to estimate *p*−values for the observed differences between the compared groups in PCoA analyses. Similarity analysis (ANOSIM) was performed through QIIME 2 software with 999 random permutations on the same Bray-Curtis distance matrix obtained from the test for differences in small-molecule repertoires among the different groups.

## Results and discussion

### Collection of a very comprehensive metagenomic shotgun dataset

A total of 6,617 publicly available shotgun metagenomic samples from 4,062 healthy infants aged between a few days of life to 4 years of life were collected and clustered according to age (Supplementary Table 1). Specifically, similar to what has been performed previously (Mancabelli et al., 2020), infant age groups were named A (*n* = 732, 0–1 month of age), B (*n* = 1,209, 1–6 months of age), C (*n* = 788, 6–12 months of age), D (*n* = 922, 12–24 months of age), and E (*n* = 411, 24–48 months of age). In order to inspect the impact of the feeding type on the developing gut microbiome, from age groups A and B, we selected 250 metagenomic fecal samples from breastfed infants and 217 from newborns fed with infant formula based on the accessible metadata associated with the published studies (Supplementary Table 1). Additionally, to generate a comprehensive dataset, a total of 2,555 shotgun metagenomic fecal samples from 18 to 70 years old human adults were retrieved from public repositories and assigned to the age group F (Supplementary Table 1).

Moreover, in order to inspect the gene expression of microbial small molecule (mSM)-related functions throughout the maturation of the human gut microbiota, we collected a dataset of 42 public metatranscriptomic samples from infants (*n* = 26, 1–6 months of age) and adults (*n* = 16).

### Species-level taxonomic profiles of microbial communities across age

The collected metagenomic shotgun sequencing data were used to longitudinally assess the relative abundance of individual gut-associated microbial species from infancy to adulthood (Supplementary Tables 2A,B). As fairly well established, the complexity and phylogenetic diversity of the infant gut microbiota progressively increase over time (Species richness, *p*-value < 0.005; Supplementary Tables 2A,B) while undergoing gradual changes in community composition toward the adult-like microbiota (PERMANOVA *p*-value of < 0.001; Supplementary Figure 1A). In particular, the milk-based diet is associated with the presence of *Escherichia coli* (average relative abundance of 9.08% at 1–6 months) and members of the *Bifidobacterium* genus such as *B. longum*, *B. bifidum*, and *B. breve* (average relative abundance of 14.64, 6.86, and 9.10%, respectively, at 1–6 months; Supplementary Figure 1B and Supplementary Table 2A), whose abundances tend to decrease following the weaning phase (4.62, 9.94, 5.04, and 5.74%, respectively, around 1 year after birth, Supplementary Table 2A). In contrast, microbial species typical of the adult gut microbiota, such as *Eubacterium rectale*, *Bacteroides uniformis*, *Bacteroides fragilis*, *Prevotella copri*, and *Faecalibacterium prausnitzii*, begin to appear with the introduction of solid foods and changes in milk consumption, reaching the highest abundance in the adulthood (Supplementary Figure 1B and Supplementary Tables 2A,B). Interestingly, fecal metagenomic data collected between 6 and 24 months after birth showed the greatest inter-individual variability among infants (C and D age groups, Supplementary Figure 1A), supporting the assumption that the weaning phase profoundly impacts the gastrointestinal environment, leading to substantial developmental adaptations of the gut microbial communities (Dizzell et al., 2021).

### Development of fecal microbial metabolic functionalities with age

**FIGURE 1:**
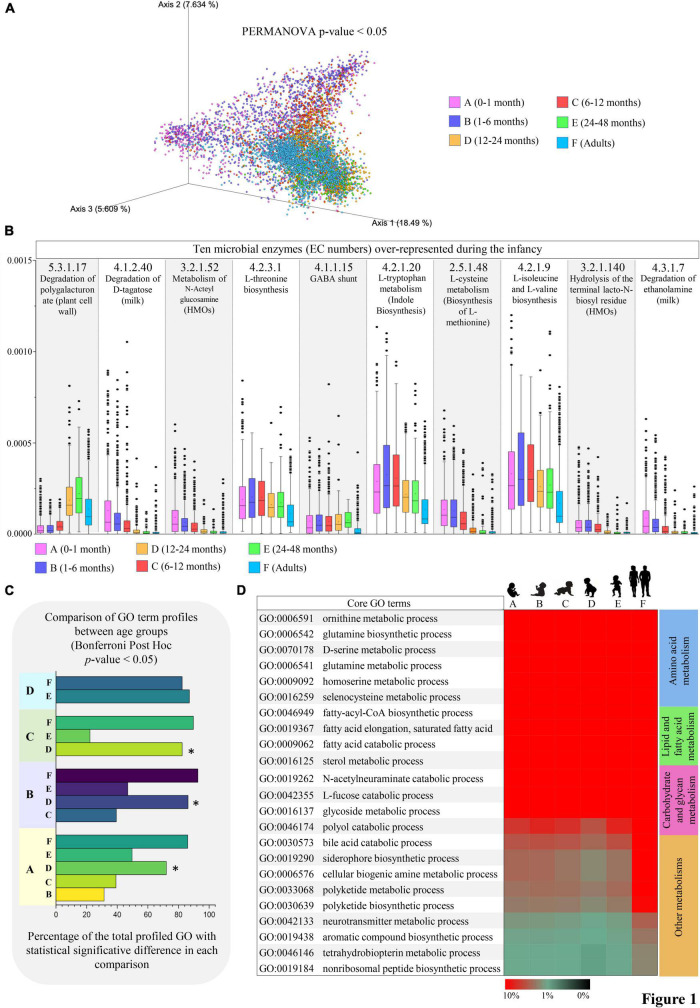
Differences in microbial pathways related to small molecule metabolisms from infancy to adulthood. **(A)** Shows the PCoA describing the age-associated differences (Bray-Curtis distances) in microbial gene profiles involved in small molecule metabolic pathways according to the MetaCyc database. **(B)** Reports unique metabolic reactions (EC number) preferentially represented in infancy. Average abundances relative to the total profiled reads are reported on the vertical axis. **(C)** Showed the number of statistically different small molecule-associated biological processes observed from pairwise comparisons between age groups. **(D)** Depicts the microbial biological processes highly represented from infancy to adulthood. Heatmap colors represent the percentage of sequenced reads assigned to a specific microbial biological process (GO term). Different colors indicate different age groups (0–1 month, pink; 1–6 months, violet; 6–12 months, red; 12–24 months, orange; 24–48 months, green; adults, light blue).

As mentioned earlier, consequently to the microbial metabolic activities, thousands of bioactive mSM can be produced at the host-microbiota interface, shaping both local and systemic host physiology and eventually influencing human health (Donia and Fischbach, 2015). In this context, the shotgun metagenomic data were used to explore the evolution of the potential functionalities related to mSM biosynthesis throughout the infant gut microbiome maturation. For this purpose, we classified the metagenomic sequenced reads according to the MetaCyc database, revealing age-associated macro-differences in the mSM metabolic profiles (PERMANOVA *p*-value < 0.05, Figure 1A and Supplementary Table 3).

Specifically, following correction for multiple comparisons (Bonferroni *Post-hoc* test *p*-value < 0.05), a total of 271 unique microbial metabolic reactions codified through Enzyme Commission (EC) numbers were identified as statistically different between the age groups (Supplementary Table 3). Notably, among the microbial metabolic activities almost exclusively present in the first year after birth (age groups A, B, and C), we found enzymes for the metabolism of milk-derived compounds, such as D-tagatose (monosaccharide; EC number 4.1.2.40), ethanolamine (amino alcohol; EC number 4.3.1.7), N-Acteylglucosamine and Lacto-N-biose (N-glycans; EC numbers 3.2.1.52 and 3.2.1.140) (Figure 1B and Supplementary Table 3). On average, their abundances progressively decreased from 0.011% in age group A to 0.0053% in age group C, in accordance with the presence of a 1-year-lasting milk-adapted microbiota (Zhou et al., 2018; Tarracchini et al., 2021b; Campbell et al., 2022). Otherwise, microbial functions involved in amino acids metabolism, including L-threonine, L-isoleucine, L-valine, and L-methionine biosynthesis (EC numbers 4.2.3.1, 4.2.1.9, and 2.5.1.48), as well as L-tryptophan catabolism with indole biosynthesis (EC number 4.2.1.20) were highly represented in the growing infants (mainly from a few days to 1 year of age) while maintaining their relevance also in the adult population (Figure 1B and Supplementary Table 3). Interestingly, this may imply that the biosynthesis of crucial microbial-derived substrates to nutrients, such as essential amino acids, is ensured at all stages of life despite the microbiome compositional changes due to age-related diet diversification (Odamaki et al., 2016).

### Gene ontology classification of the enzymatic repertoire involved in microbial small molecule metabolism

In order to gain an overview of the age-associated variation in microbial biological processes, we classified the profiled microbial enzyme-encoding genes according to the Gene Ontology (GO) system (Supplementary Table 4; Harris et al., 2008).

Notably, by comparing the obtained microbial functional repertoires between age groups, we observed that age group D (12–24 months of age) showed the highest number of statistically significant GO terms in each comparison (ANOVA, *p*-value < 0.05; Figure 1C), thus diverging from the patterns observed in any other infancy time-point (Figure 1C). This may describe the dramatic changes in the microbial community composition undertaken concurrently with the cessation of milk intake. Indeed, it has been argued that the conclusion of milk-based diet (breast milk and/or formula) rather than the introduction of solid food at around 6 months of age induces profound modifications in the infant gut microbiome structure, leading it toward an adult-like state (Davis et al., 2017).

However, among the first 20 more abundant mSM core metabolism, i.e., microbial biological processes (GO terms) highly represented from birth to adulthood, we identified functions related to the production and degradation of fatty acids, biogenic amines (many of which act as eukaryotic neurotransmitters), glutamine, polyketides, and non-ribosomal peptides (which exhibit narrow-spectrum antimicrobial activities), aromatic amino acids (L-tryptophan, L-tyrosin, and L-phenylalanine), and siderophores, along with specific functions for the catabolism of bile acids, L-fucose, and N-acetylneuraminate (sialic acid) (Figure 1D). Remarkably, these activities appear crucial for intestinal niche colonization by mediating bacterial competition, quorum sensing, and the utilization of the available carbohydrate sources, including human milk oligosaccharides and mucin (Spano et al., 2010; Zimmermann and Fischbach, 2010; Yu et al., 2013; Donia and Fischbach, 2015; Pickard and Chervonsky, 2015; Guzior and Quinn, 2021).

**FIGURE 2:**
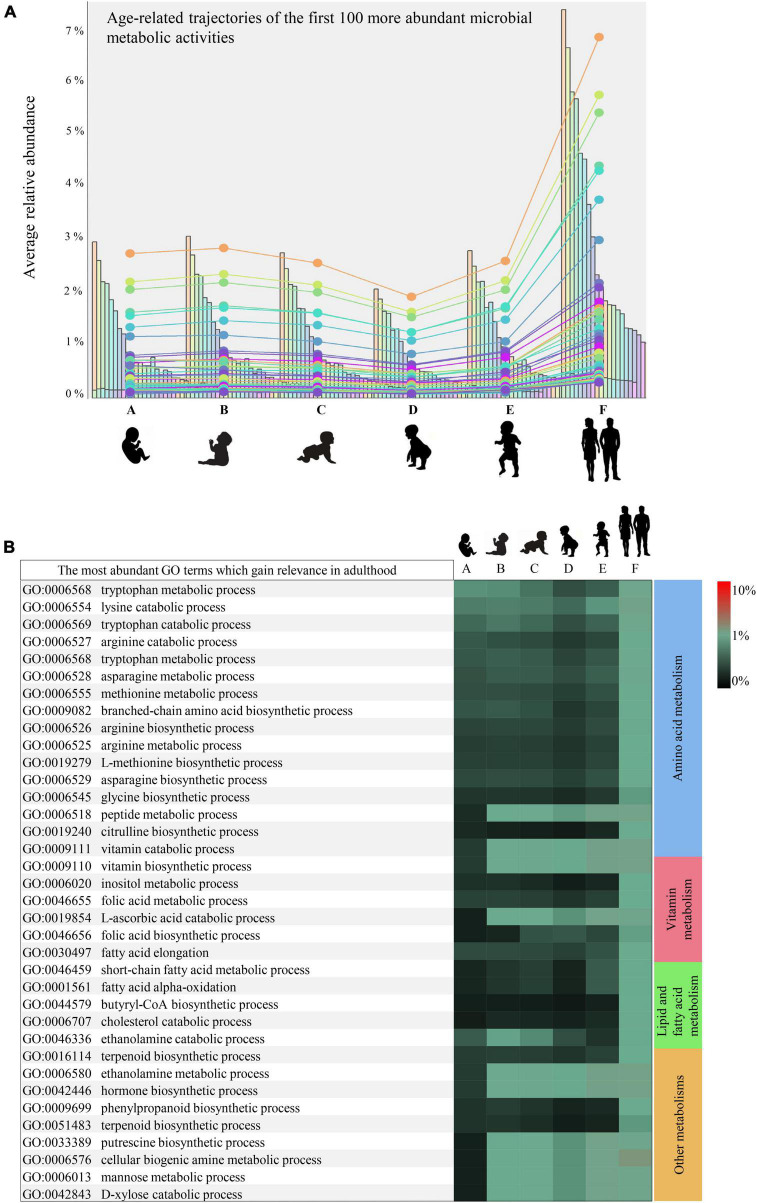
Developmental trajectories of the microbial small-molecule-related functions. **(A)** Shows the developmental trend in the relative abundance of the small molecule-related microbial functions. In **(B)**, relative abundances of the leading small molecule metabolisms enriched in adulthood rather than infancy are illustrated by a heatmap. Heatmap color scale illustrates the percentage of sequenced reads assigned to a specific microbial biological process (GO term). Different letters indicates different age groups (0–1 month, A; 1–6 months, B; 6–12 months, C; 12–24 months, D; 24–48 months, E; adults, F).

Furthermore, as exemplified in Figure 2A reporting only the first 100 more abundant microbial-derived GO terms of biological processes, nearly all (84%) of the statistically significant mSM-associated microbial activities profiled in adulthood (minimum abundance 0.05%) were already present in 1-month-old infants, albeit with a significantly lower abundance (minimum abundance 0.03%, ANOVA *p*-value < 0.05) (Supplementary Table 4). Thus, highlighting an overall progressive enhancement of the early established microbial metabolic potential over time.

Specifically, synthesis or utilization of (branched-chain, sulfur, and aromatic) amino acids, biogenic amines such as putrescine, and vitamins appeared 1 month after birth with abundances ranging from 0.02 to 0.07% and then significantly enriched in adulthood (abundances between 0.05 and 0.19%) (Bonferroni *Post-hoc* test *p*-value < 0.05) (Figure 2B). In contrast, synthesis pathways of butyric acid, terpenoids, and non-protein amino acids such as citrulline, along with metabolisms of cholesterol, mannose, and xylose, were found among the small molecule metabolisms nearly uniquely present in adulthood (Figure 2B).

All the microbial functions found preferentially associated with adulthood are believed to provide several metabolites relevant for the microbe-host mutualisms, contributing positively to the host physiology. In particular, butyric acid and polyamines, including putrescine, have demonstrated beneficial effects on the human gut mucosa (Tofalo et al., 2019), while vitamins and various amino acids can translocate in systemic circulation and exert far-reaching effects on the host (Magnúsdóttir et al., 2015; van der Wielen et al., 2017). Therefore, the infant gut microbiota maturation emerged to be far from concluded even at 3–4 years of age, albeit the taxonomic composition may resemble that of adults (Zeng et al., 2022).

However, from about 1 month after birth, infant gut-associated microbial communities appeared genetically equipped with most of the microbial metabolic functions that will support intestinal homeostasis and physiological processes in adults, suggesting a very early foundation of the host-microbiota symbiosis, with improvements in the microbial metabolic potential throughout the host development. These data indicate that host-microbiome co-evolution led to the selection of microbial genetic traits necessary for survival and growth in the varying intestinal niche as well as for the perpetual production of small molecules of high biological relevance in the host physiology.

### Early infant feeding practices shape the metabolic traits of the fecal microbial communities

It is very well known that the feeding type is among the key factors influencing the infant gut microbiota composition and, therefore, gastrointestinal functions (Yatsunenko et al., 2012). In order to evaluate the gut microbiome structure as a function of the infant diet (breastfeeding vs. formula feeding), we considered 250 shotgun metagenomic samples from breastfed newborns and 217 from formula-fed infants from the age group A (0–1 month) and B (1–6 months) (Supplementary Table 1). As highlighted in Supplementary Figure 2A, except for the 0–1 month-old infants showing high inter-individual variability, infant diet is associated with distinct gut microbial compositional patterns (PERMANOVA *p*-value < 0.05; Supplementary Figure 2A), with significantly lower growth of *E. coli* throughout the lactation and higher abundance of *B. breve* and *B. bifidum* at 1–6 months in breastfed infants rather than in those fed with infant formula (*t*-test *p*-value < 0.05) (Supplementary Figure 2B).

**FIGURE 3:**
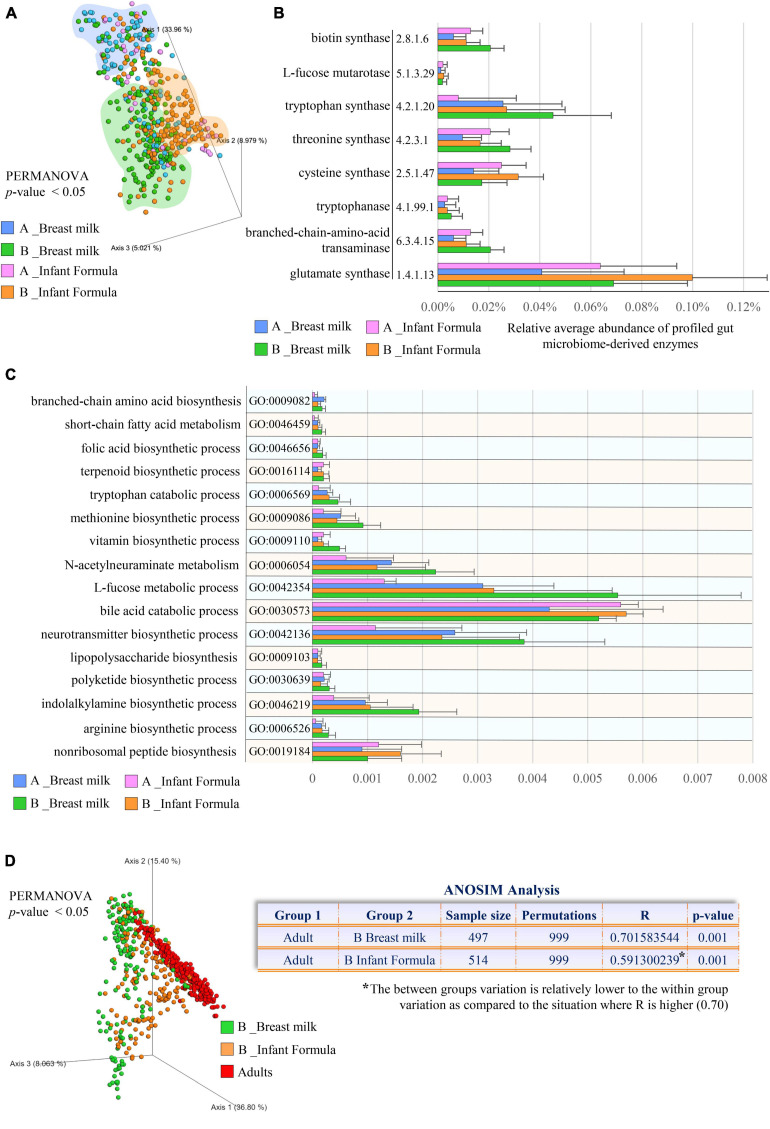
Comparison of the microbial small molecule profiles between metagenomes of breastfed and formula-fed infants. In **(A)**, the PCoA depicts the significant differences in the small molecule profiles between breastfed and formula-fed infants aged 0–1 months (A) and 1–6 months (B). **(B)** Exhibits the microbial small molecule-related pathways (EC number) with statistically significant differences between feeding types. **(C)** Shows differentially abundant microbial biological processes involved in small molecule metabolisms classified according to the Gene Ontology (GO) annotation system. In **(D)**, PCoA and ANOSIM analysis compare the small molecule repertoires observed in different feeding practices (breastfeeding and formula) and adults. Different colors indicate different ages and feeding types (0–1 month old breast fed, light blue; 1–6 months old breast fed, green; 0–1 month old formula fed, pink; 1–6 months old formula fed, orange).

Classification of the enzyme-encoding genes using the MetaCyc database of small molecules, combined with functional enrichment based on the GO annotation system, revealed microbial functional diversity according to the feeding type (PERMANOVA *p*-value < 0.05; Figure 3A). In particular, compared with formula-fed infants, those receiving breast milk were enriched in microbial metabolisms involved in the degradation of L-fucose (GO:0042354), (GO:0030573), and N-acetylneuraminate (GO:0006054), along with biosynthetic pathways of indole-3-acetic acid (L-tryptophan metabolism, GO:0006569), and polyketides (GO:0030639) (Figures 3B,C and Supplementary Tables 5, 6). Moreover, microbial production of vitamins, including folic acid (vitamin B9, GO:0046656) and biotin (vitamin B7; EC number 6.3.4.15), became significantly preponderant in breastfed infants 1–6 months after birth (Figure 3B and Supplementary Table 5). This could explain why, although human breast milk contains a slightly low concentration of biotin, no signs of biotin deficiency were noted in breastfed newborns (Yatsunenko et al., 2012).

Similarly, breastfed infants showed an increase in microbial biological processes involved in the biosynthesis of L-arginine (GO:0006526), L-methionine (GO:0009086), L-tryptophan (EC number 4.2.1.20), L-threonine (EC number 4.2.3.1), L-cysteine (EC number 2.5.1.47), L-glutamate (EC number 1.4.1.13), and branched-chain amino acids (GO:0009082) (Bonferroni *Post-hoc* test *p*-value < 0.05) (Figures 3B,C and Supplementary Table 6), corresponding to the amino acids with a lower concentration in breastmilk (Zhang et al., 2013). More specifically, while the human milk content of these latter amino acids declines with lactation progression (Zhang et al., 2013), we found that their microbial production tends to increase, completing breast milk composition throughout the natural dynamic changes of lactation, thus supporting the high protein requirement in developing infants (Garlick, 2006; Ballard and Morrow, 2013).

Beyond the specific microbial activities that we found enriched in breastfed infants, microbial functional analyses highlighted that most (64%) of the profiled GO terms were more abundant in formula-fed infants, indicating that compared with breastfed newborns, the gut microbiome of infants receiving formula resembles earlier that of adults (ANOSIM *R* = 0.59 vs. *R* = 0.70 at *p*-value = 0.001; Figure 3D and Supplementary Table 6). Accordingly, this evidence suggested that formula-fed infant gut microbiota may evolve more rapidly compared with that of their breastfed counterpart, as previously partially observed solely from a taxonomic point of view through the use of specific metrics, i.e., “relative microbiota maturity” and “microbiota-for-age Z score” (Bäckhed et al., 2015; Stewart et al., 2018). Thus, lack of breast milk intake could preclude the gradual specialization of the gut microbiota that instead parallelly accompanies the growth of the breastfed newborn with the well-known positive effects on intestinal, neurological, and immune system development (Guaraldi and Salvatori, 2012; Jost et al., 2012; van den Elsen et al., 2019).

Altogether, these findings widen the repertoire of the notorious benefits of breastfeeding to the production of bioactive mSM with relevant biological roles in the host.

### Changes in the metatranscriptome profiles driving small molecule metabolisms along the infant gut microbiome development

**FIGURE 4:**
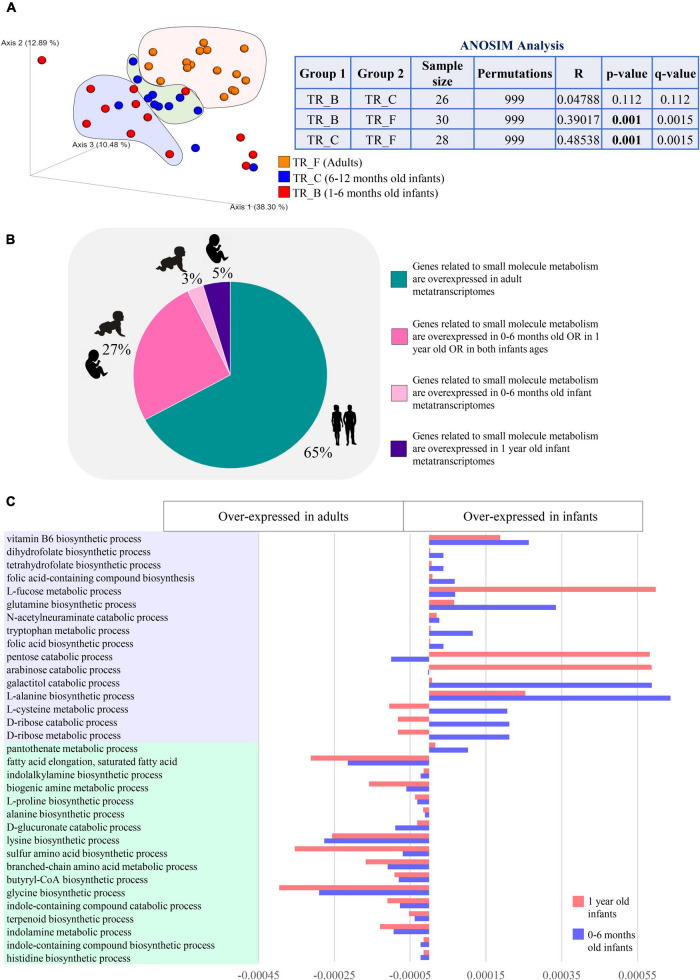
Differences in the expression of microbial functions related to small molecule metabolism based on metatranscriptome level analysis. In **(A)**, macro differences in the small molecule gene expression patterns between infants and adults are described through PCoA and ANOSIM analysis. Different colors indicate different ages groups (1–6 months old, TR_B, red; 6–12 months old, TR_C, blue; and adults, TR_F, orange). In **(B)**, pie chart depicts the distribution among infant and adult metatranscriptomes of the differentially expressed genes encoding small molecule-related functions. Different colors indicate genes overexpressed in different age groups (genes overexpressed in adults, green; genes overexpressed in 0–6 months old OR in 1 year old OR in both infants ages, dark pink; genes overexpressed in 0–6 months old infants, light pink; genes overexpressed in 1 year old infants, violet). **(C)** Reports developmental changes in the expression of selected small molecule-related metabolisms.

In order to inspect expression patterns of microbial genes related to the mSM metabolism in the human gut microbiota from infancy to adulthood, we collected a total of 42 public available cross-sectional metatranscriptomic samples from infants aged between 1 month and 1 year along with adults (18–70 years) (Supplementary Table 1). According to age, the datasets were then gathered in age groups TR_B (*n* = 14, 1–6 months old), TR_C (*n* = 12, 6–12 months old), and TR_F (*n* = 16, adults). Similarity analysis (ANOSIM) and visualization of the variation dispersal at the metatranscriptome level (PCoA) showed that differences among samples within infant age groups TR_B and TR_C (including those resulting from different delivery routes and feeding types) were lower than those emerging by comparison with TR_F, suggesting that age-related factors are the main forces driving the maturation of the gut metatranscriptome (Figure 4A). Overall, the metatranscriptome-level analysis revealed that 67% of mSM-related microbial activities were overexpressed in adults, implying that the reduced genetic potential for microbial functionality characterizing the infant gut microbiome, described above compared to adults, is also reflected at the metatranscriptome level (Figure 4B and Supplementary Table 7). Specifically, adult-like metatranscriptomes showed increased expression of microbial biological processes involved in a wide range of amino acid metabolisms, including branched-chain and sulfur-containing amino acids, as well as L-lysine, L-histidine, and L-proline (Figure 4C and Supplementary Table 7). Besides, also functions related to the metabolism of fatty acids, biogenic amines, terpenoids, and indole-containing compounds, as well as butyric acid biosynthesis, were significantly over-expressed in adulthood (Figure 4C and Supplementary Table 7), thus, evidencing a wide variety of mSM related activities with a progressive evolution of their genes’ expression patterns over time.

In contrast, infants showed the over-expression of biological processes involved in the catabolism of L-tryptophan, along with biosynthetic pathways of several vitamins, including B9, B5, B6, and K2, L-glutamine, L-methionine, L-cysteine, and L-homoserine, which acts as a precursor for methionine, threonine, and isoleucine (Figure 4C and Supplementary Table 7). Also, degradation of monosaccharides, such as D-ribose, D-arabinose, D-glucose, D-galactose, and pentoses, along with pathways for the utilization of L-fucose and N-Acetylneuraminic acid, were overexpressed in infancy compared to adulthood, suggesting overall an active biosynthetic metabolism that relies more on simple diet-derived sugars and host-derived oligosaccharides (Figure 4C and Supplementary Table 7).

Interestingly, 65% of the mSM-related microbial metabolisms over-expressed in infancy did not show significant abundance differences between infants and adults at the metagenome/gene level, while the remaining 35% of infant over-expressed genes were among those we found less abundant in the metagenome of infants rather than adults. Altogether, these findings confirmed that developmental stage-specific mSM-associated microbial functions are accomplished through the accommodation of gene expression of the existing genetic metabolic potential rather than solely through rearrangements of the microbial genetic makeup.

## Conclusion

Under homeostatic conditions, the gut microbiota metabolizes the diet- and host-derived substrates available in the intestinal environment, producing a multitude of bioactive metabolites, such as small molecules, with positive impacts on host physiology (Luber and Kostic, 2017). However, developmental adjustments in microbial activities related to the small molecule metabolism in correlation with age remain poorly investigated.

In this context, analyses of 6,617 shotgun metagenome fecal samples from infants and adults highlighted that 4-years old infants still showed underdeveloped microbial activities compared to adults. This implies that although the infant gut microbiota is considered compositionally mature around 3 years after birth, developmental processes of small molecule-associated functionalities are still far from accomplished. However, as highlighted from the metatranscriptome perspective, most microbial genetic features for small molecule metabolisms that are highly expressed in adulthood were already present at 1 month of life, although with a lower abundance. Similarly, a portion of the small molecule-related functions overexpressed in infancy corresponded to those with a lower abundance compared to adult metagenomes. This fact points to a fine modulation of the microbial small molecule-related activities through gene expression patterns that contribute to meeting stage-specific host physiological demands.

Furthermore, comparison of different infant feeding practices (breastfeeding vs. infant formula) showed that breastfed infants were enriched in microbial pathways involved in the biosynthesis of amino acids with low concentration in human milk, pointing out the existence of complementarity between breast milk and gut microbiome functionalities.

Altogether, these data highlighted that the close cooperation between gut bacteria and host cells relies on the early establishment of the microbial metabolic (genetic) potential, whose expression will be dynamically adapted in response to the availability of nutritive resources as well as the physiological needs of the specific host’s developmental stages. In this regard, findings collected in this meta-analysis will be pivotal for future studies aimed at comparing infant small molecule profiles in different nutritional conditions as well as identifying the major gut commensals producers of such bioactive metabolites.

## Data availability statement

The original contributions presented in this study are included in the article/Supplementary material, further inquiries can be directed to the corresponding author/s.

## Author contributions

FT, MV, and CM: conceptualization, supervising, coordination, and revising of the manuscript. CT, FF, GL, LM, and GA: data collection and analyses. CT and CM: drafting of the manuscript. All authors contributed to the article and approved the submitted version.

## Footnotes

1
https://www.ncbi.nlm.nih.gov/refseq/

2
https://www.originlab.com/2021

3
https://bioconductor.org/packages/GO.db/

4
www.ibm.com/software/it/analytics/spss/

5
https://www.originlab.com/

## Supplementary material

Supplementary Figure 1 Differences in the gut microbiota composition from infancy to adulthood. In **(A)**, macro-differences in the composition of the gut microbiomes from infants (0–4 years) and adults are visualized through the PCoA plot. **(B)** Reports selected microbial taxa with significant differences in abundance from infancy toward adult-like composition.

Supplementary Figure 2 Differences in the gut microbiota composition between different infant feeding types (breastfeeding vs. formula). **(A)** Shows the PCoA representing the differences in microbial taxonomic composition between breastfed and formula-fed infants aged 0–1 month (A) and 1–6 months (B). **(B)** Shows microbial species with significantly different profiles depending on the infant feeding. Heatmap color scale depicts the average relative abundance of the statistically significant microbial species in different feeding types. Different letters (A–F) and colors are used to identify life stages and infant feeding practices.
