# Supplementary figures and images for: Gut microbe metabolism of small molecules supports human development across the early stages of life
Source: Front Microbiol. 2022 Sep 13;13:1006721. doi: 10.3389/fmicb.2022.1006721 (PMC9512645; doi:10.3389/fmicb.2022.1006721)

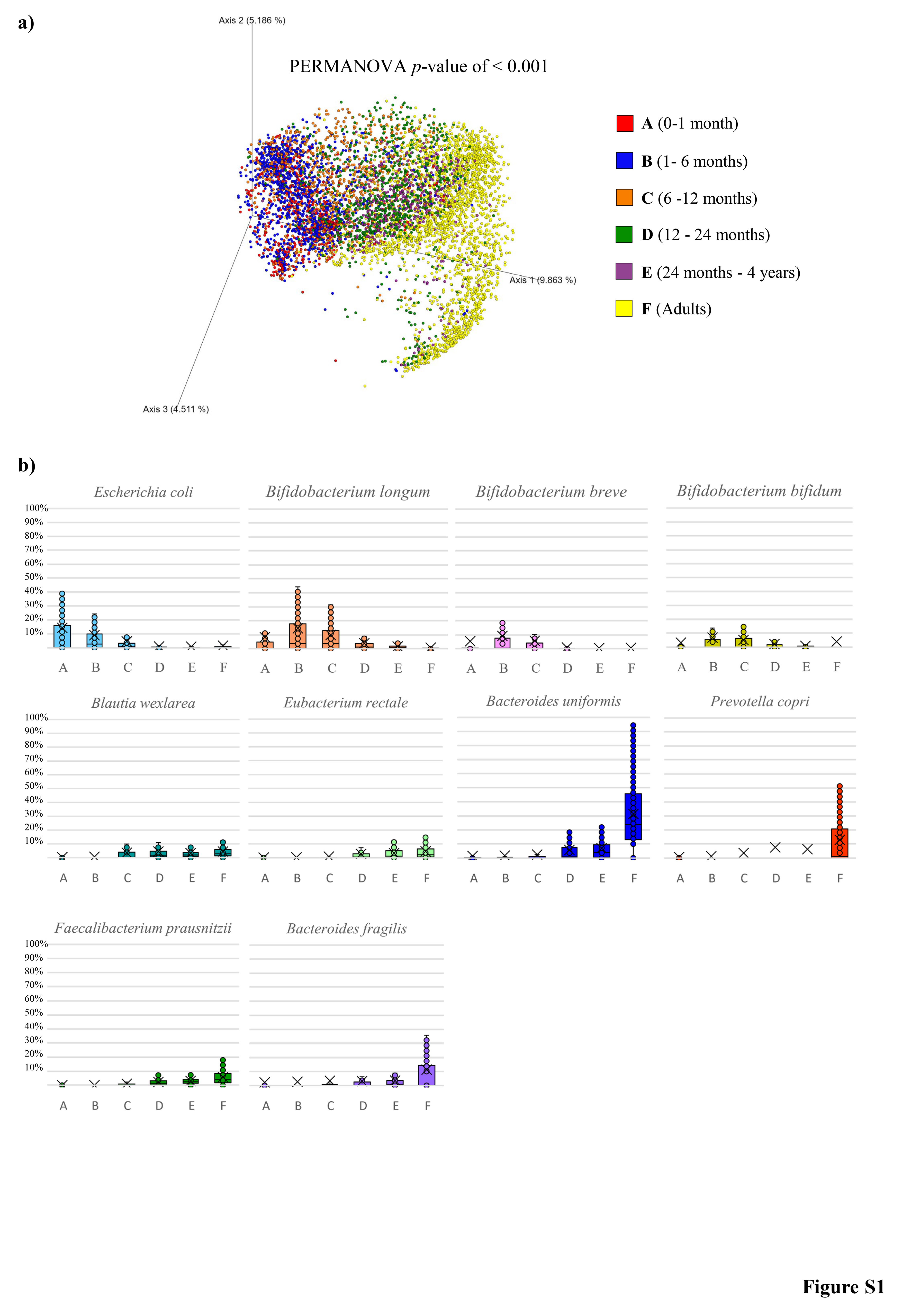

Supplement: Supplementary Figure 1 — Differences in the gut microbiota composition from infancy to adulthood. In (A), macro-differences in the composition of the gut microbiomes from infants (0–4 years) and adults are visualized through the PCoA plot. (B) Reports selected microbial taxa with significant differences in abundance from infancy toward adult-like composition. [file Image_1.TIF]

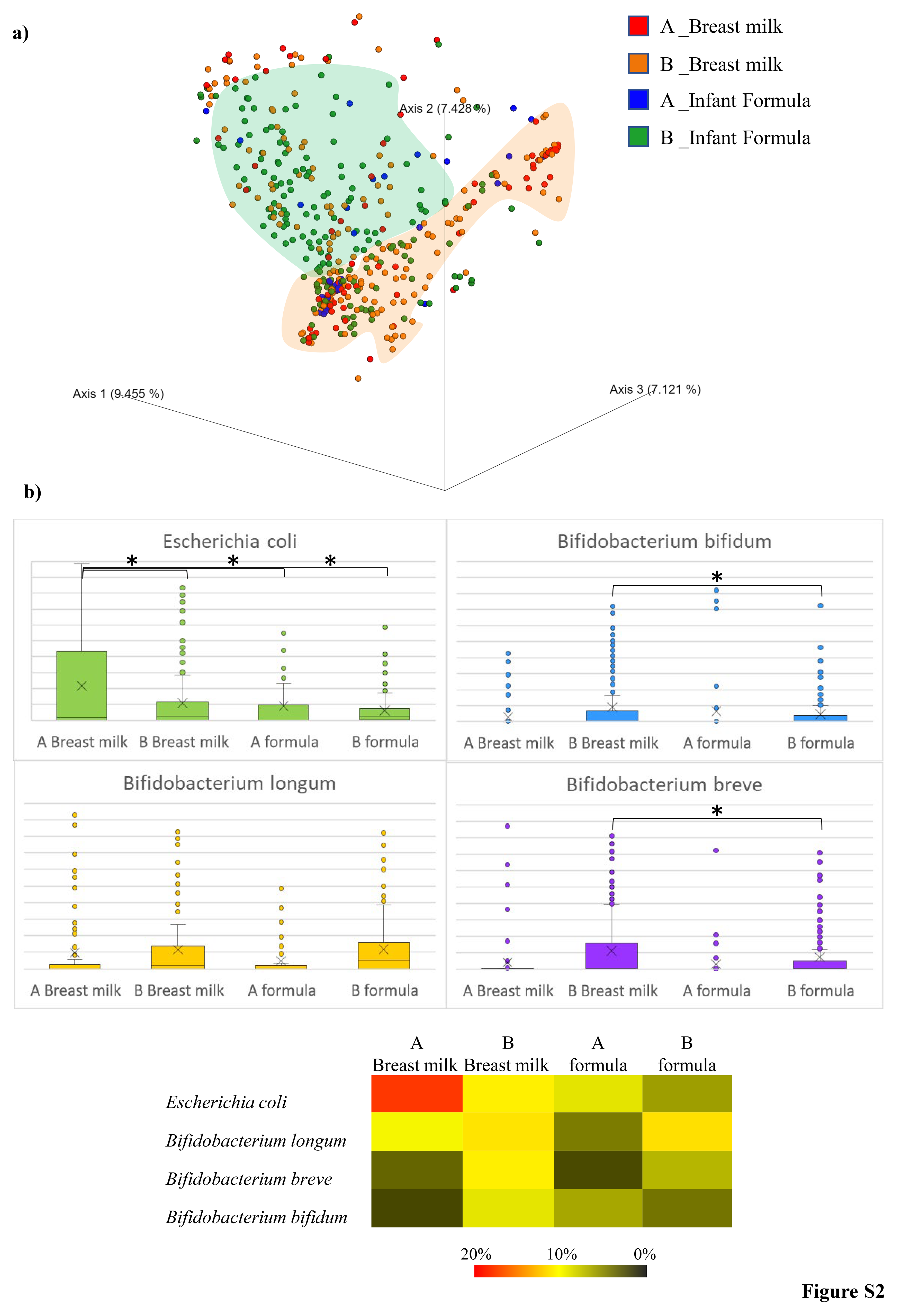

Supplement: Supplementary Figure 2 — Differences in the gut microbiota composition between different infant feeding types (breastfeeding vs. formula). (A) Shows the PCoA representing the differences in microbial taxonomic composition between breastfed and formula-fed infants aged 0–1 month (A) and 1–6 months (B). (B) Shows microbial species with significantly different profiles depending on the infant feeding. Heatmap color scale depicts the average relative abundance of the statistically significant microbial species in different feeding types. Different letters (A–F) and colors are used to identify life stages and infant feeding practices. [file Image_2.TIF]
